# Copy Number Heterogeneity, Large Origin Tandem Repeats, and Interspecies Recombination in Human Herpesvirus 6A (HHV-6A) and HHV-6B Reference Strains

**DOI:** 10.1128/JVI.00135-18

**Published:** 2018-04-27

**Authors:** Alexander L. Greninger, Pavitra Roychoudhury, Negar Makhsous, Derek Hanson, Jill Chase, Gerhard Krueger, Hong Xie, Meei-Li Huang, Lindsay Saunders, Dharam Ablashi, David M. Koelle, Linda Cook, Keith R. Jerome

**Affiliations:** a Department of Laboratory Medicine, University of Washington, Seattle, Washington, USA; b Fred Hutchinson Cancer Research Institute, Seattle, Washington, USA; c Department of Medicine, University of Washington, Seattle, Washington, USA; d Department of Global Health, University of Washington, Seattle, Washington, USA; e HHV-6 Foundation, Santa Barbara, California, USA; f Department of Pathology and Laboratory Medicine, University of Houston, Houston, Texas, USA; Northwestern University

**Keywords:** human herpesvirus 6, HHV-6, HHV-6A, HHV-6B, copy number, secondary standards, Z29, GS, origin of replication, origin, tandem repeat, nanopore, nanopore sequencing, direct repeat, quantitative PCR, international standard, reference materials

## Abstract

Quantitative PCR is a diagnostic pillar for clinical virology testing, and reference materials are necessary for accurate, comparable quantitation between clinical laboratories. Accurate quantitation of human herpesvirus 6A/B (HHV-6A/B) is important for detection of viral reactivation and inherited chromosomally integrated HHV-6A/B in immunocompromised patients. Reference materials in clinical virology commonly consist of laboratory-adapted viral strains that may be affected by the culture process. We performed next-generation sequencing to make relative copy number measurements at single nucleotide resolution of eight candidate HHV-6A and seven HHV-6B reference strains and DNA materials from the HHV-6 Foundation and Advanced Biotechnologies Inc. Eleven of 17 (65%) HHV-6A/B candidate reference materials showed multiple copies of the origin of replication upstream of the U41 gene by next-generation sequencing. These large tandem repeats arose independently in culture-adapted HHV-6A and HHV-6B strains, measuring 1,254 bp and 983 bp, respectively. The average copy number measured was between 5 and 10 times the number of copies of the rest of the genome. We also report the first interspecies recombinant HHV-6A/B strain with a HHV-6A backbone and a >5.5-kb region from HHV-6B, from U41 to U43, that covered the origin tandem repeat. Specific HHV-6A reference strains demonstrated duplication of regions at U1/U2, U87, and U89, as well as deletion in the U12-to-U24 region and the U94/U95 genes. HHV-6A/B strains derived from cord blood mononuclear cells from different laboratories on different continents with fewer passages revealed no copy number differences throughout the viral genome. These data indicate that large origin tandem duplications are an adaptation of both HHV-6A and HHV-6B in culture and show interspecies recombination is possible within the Betaherpesvirinae.

**IMPORTANCE** Anything in science that needs to be quantitated requires a standard unit of measurement. This includes viruses, for which quantitation increasingly determines definitions of pathology and guidelines for treatment. However, the act of making standard or reference material in virology can alter its very accuracy through genomic duplications, insertions, and rearrangements. We used deep sequencing to examine candidate reference strains for HHV-6, a ubiquitous human virus that can reactivate in the immunocompromised population and is integrated into the human genome in every cell of the body for 1% of people worldwide. We found large tandem repeats in the origin of replication for both HHV-6A and HHV-6B that are selected for in culture. We also found the first interspecies recombinant between HHV-6A and HHV-6B, a phenomenon that is well known in alphaherpesviruses but to date has not been seen in betaherpesviruses. These data critically inform HHV-6A/B biology and the standard selection process.

## INTRODUCTION

Human herpesvirus 6B (HHV-6B) is a ubiquitous human virus with human exposure levels of >90% by the age of 2 years as measured by serological assays performed worldwide (1, 2). Both HHV-6A and HHV-6B establish chronic infections in the majority of infected individuals, leading to asymptomatic persistent viral shedding (3, 4). Exanthema subitum is the most common HHV-6B-related infection seen after a primary exposure in 6-month- to 3-year-old children (5). Less frequently the virus can result in seizures, gastrointestinal and respiratory symptoms, thrombocytopenia, hepatitis, colitis, and central nervous system (CNS) infections (6). Additionally, both HHV-6A and HHV-6B have been shown to integrate into host chromosomes in the telomere regions and to be passed from parents to their children as inherited chromosomally integrated HHV-6A/B (iciHHV-6A/B) (7–9). The potential to detect HHV-6A/B in genomic DNA from these patients, as well as possible reactivation from the integrated HHV-6A/B, makes the diagnosis of HHV-6A/B infection from serum or plasma viral load testing challenging (10, 11).

Several prototypic strains of HHV-6A/B have been identified and utilized, including GS, U1102, SIE, LHV, Z29, and HST (12–16). Until very recently, there were fewer than 200 HHV-6A/B sequences in GenBank, including only 3 complete genomes. HHV-6A and -6B genomes have approximately 90% nucleotide identity to each other and about 50% similarity to the most closely related human betaherpesvirus, HHV-7. The genome is approximately 160 to 170 kb and contains many of the gene and regulatory elements present in the genomes of other betaherpesviruses. Despite the close sequence identity between HHV-6A and -6B, no interspecies recombinants have been isolated in nature to date. In comparison, interspecies herpes simplex virus 1 (HSV-1) and HSV-2 recombination is relatively common, even though they share significantly less sequence homology (17, 18). Recently, two large-scale efforts have sequenced more than 150 nearly full-length HHV-6B genomes from four continents to add to existing HHV-6A/B genomes (19, 20).

Accurate and sensitive real-time PCR assays that detect and quantify HHV-6A/B are critical to diagnosis and monitoring of the variety of manifestations of HHV-6A/B-associated disease. Recently, several quantitative cutoffs have been proposed that are associated with end-organ disease or iciHHV-6A/B status (21–23). A review of the PCR methods used in 46 recently published papers (2014 to 2017) revealed the use of 17 different primer sets at multiple locations throughout the genome (locations U6, U12, U13, U22, U27, U31, U32, U38, U41, U57, U65, U66, U67, U69, U90, U95, and U100), with only the U31, U65, and U66 primers used more than twice. Not surprisingly, cross-laboratory proficiency testing has shown differences in quantitation as high as 4 log units (24, 25). Previous studies have identified the critical role that standardized materials play in the ability to establish clinical viral load cutoffs and assay sensitivity and to compare results between laboratories (24, 26–30). Finally, effective primer designs have been significantly limited by the lack of available DNA sequences.

Previous work has reported linear amplification of the lytic origin of replication of HHV-6B strain Z29 to be associated with increased viral passage and increased replication *in vitro* (31, 32). In an effort to determine whether the available cultured “reference” strains have undergone significant changes during culture similar to that seen with the recently produced WHO BK and JC strains, we obtained 15 strains from the HHV-6 Foundation repository and used shotgun sequencing to obtain full-length genomes and estimates of copy numbers (33, 34). We found 9 of the 15 strains had high-copy-number tandem-repeat amplifications in the origin of replication, including the above-mentioned HHV-6B strain Z29, albeit with different breakpoints than previously described, as well as the first described origin amplification in an HHV-6A strain. We also describe the first HHV-6A/HHV-6B interspecies recombinant. Other HHV-6A/B reference materials had multiple loci with copy number variations of up to 20 times. These duplications, deletions, and rearrangements may impact the utility of the strains for the production of standard materials for PCR testing. Changes in the genomes of the strains in culture may have an impact on the results of current and future studies utilizing these materials.

## RESULTS

### Large tandem repeats covering the origin of replication in both HHV-6A and HHV-6B strains.

**FIG 1:**
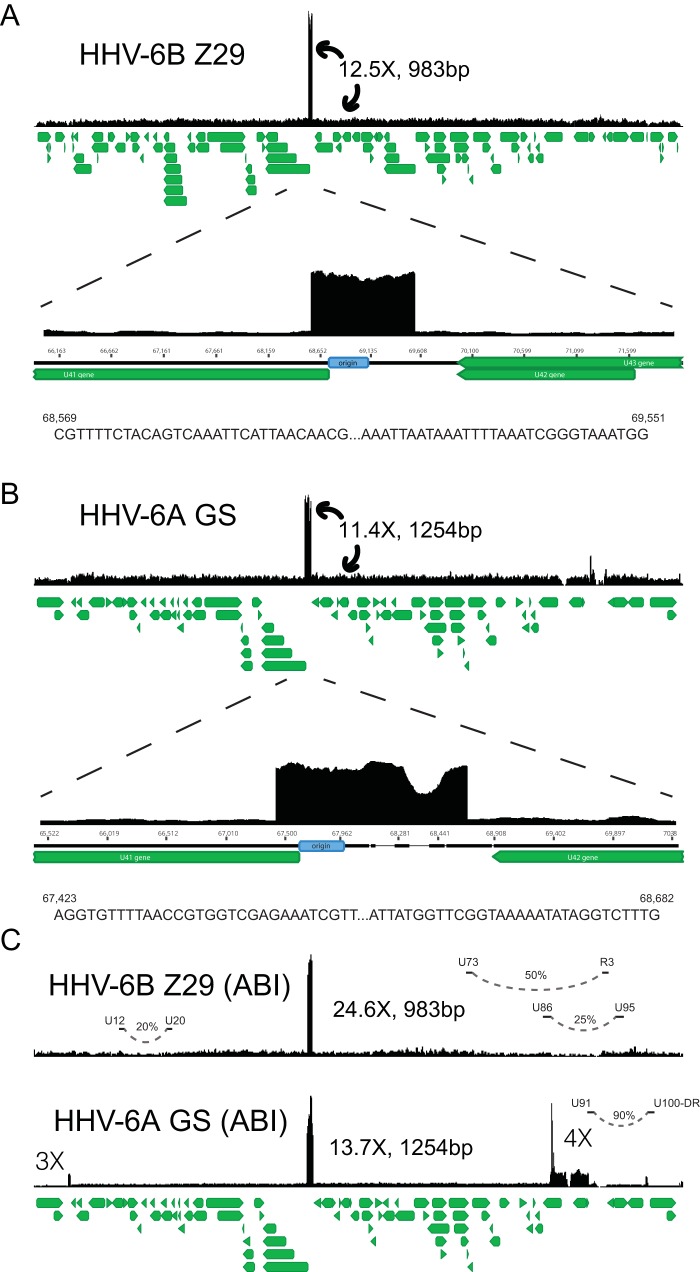
Representative coverage maps of HHV-6B Z29 and HHV-6A GS reference strains. Shotgun DNA-sequencing reads from cultured virus were mapped to the NCBI HHV-6B and HHV-6A reference genomes, NC_000898 and NC_001664, respectively. The green stacked lines indicate the gene models for the respective viral species. (A) HHV-6B strain Z29 yielded a homogeneous 983-bp tandem repeat that was present at approximately 12.5 times higher coverage than the rest of the genome. Sequences at the 5′ and 3′ ends of the tandem repeat in strain Z29 are depicted and are different than those indicated previously (31). (B) HHV-6A strain GS yielded a heterogeneous 1,254-bp tandem repeat that was present at approximately 11.4 times higher coverage than the rest of the genome. Sequences at the 5′ and 3′ ends of the heterogeneous tandem repeat in strain GS are depicted. (C) ABI quantitative DNA material for HHV-6A GS and HHV-6B Z29 also demonstrated similar origin tandem repeats with additional loci with copy number differences in the GS strain. Long-distance rearrangements between U12 to U20, U73 to R3, U86 to U95, and the U91-to-U100/DR intergenic region are represented by curved dashed lines, and the estimated viral subpopulation containing the respective deletion is indicated by the percentage.

In order to obtain single nucleotide resolution and copy number measurement for HHV-6A/B type strain reference materials, we sequenced an HHV-6A GS strain obtained from the HHV-6 Foundation and a HHV-6B Z29 strain obtained from the NIH AIDS repository. Libraries of the Z29 and GS strains were each prepared twice and sequenced to an average depth of 76× and 453×, respectively. The HHV-6B Z29 strain contained a homogeneous 983-bp-long tandem repeat (Fig. 1A). Copy number estimates based on relative coverage at the edges of the repeats across multiple library preparations indicated an average of 11 to 13 copies of the repeat were present. Mapping of the edges of the Z29 origin tandem repeat gave different repeat breakpoints than previously described for what was possibly a different passage lineage (31). All the Z29 strains sequenced in this study had an additional 123 nucleotides at the 5′ end of the repeat and an extra 4 nucleotides at the 3′ end of the repeat compared to the previously described repeat to make the 983-bp tandem repeat (31).

HHV-6A strain GS contained a heterogeneous tandem repeat that covered 1,254 bp of the HHV-6A reference genome (Fig. 1B). Copy number estimates based on relative coverage at the edges of the repeats indicated an average of 10 to 12 copies of the repeat were present. The most common tandem repeat present included deletions of 193 bp and 2 bp, along with an insertion of 189 bp, based on the HHV-6A reference genome, giving a mode length of 1,254 bp.

**FIG 2:**
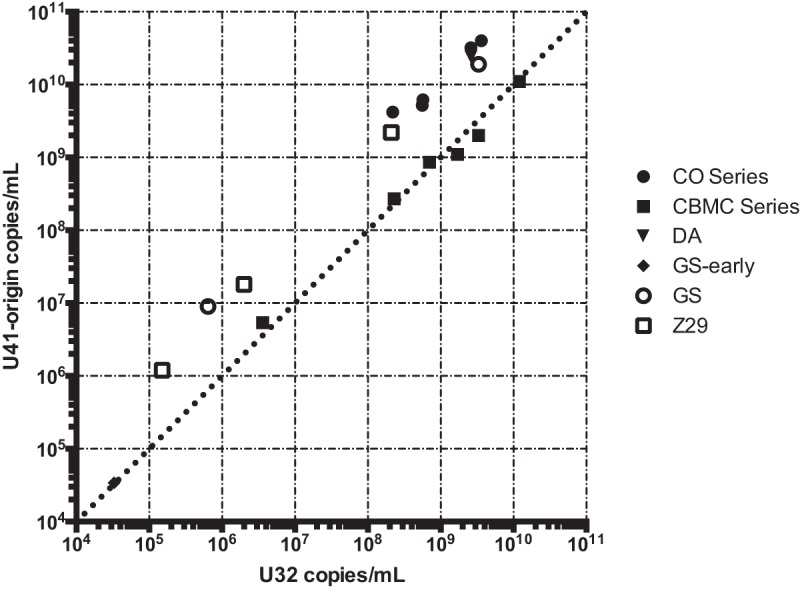
qPCR analysis of U32 and origin loci for DNA from HHV-6A/B strains confirming deep-sequencing estimates of origin copy numbers. The plotted quantities are listed in Table 1.

To demonstrate that the copy number heterogeneity we found in the type strains of HHV-6A/B are also present in commercially available quantitative clinical reference materials, we also performed shotgun DNA sequencing on an HHV-6A GS strain and an HHV-6B Z29 strain from Advanced Biotechnology Inc. (ABI). These strains had tandem repeats that were similar in size at the origin of replication present in the GS strain obtained from the HHV-6 Foundation and Z29 strains obtained from the NIH AIDS repository (Fig. 1C). However, the Z29 origin tandem repeat in the commercial reference material was present at approximately twice the copy number observed in Z29 from the NIH AIDS repository. Intriguingly, the HHV-6A GS strain quantitative secondary standard material also contained a 4-fold increase in coverage, covering the U90 and U91 genes and the N-terminal two-thirds of the U86 gene. The U91 end of the repeat contained a complex rearrangement with the U100-direct repeat (DR) intergenic region 266 nucleotides 5′ of the beginning of the annotated DR repeat region. The HHV-6B Z29 secondary standard contained three large rearrangements associated with the presence of junctional reads— one between U12 and U20 constituting ∼20 to 30% of the genomes present, one between U73 and R3 repeat region constituting ∼50% of the genomes present, and one between U86 and U95 representing ∼25% of the genomes present. Thus, the copy number of HHV-6B between U86 and R3 was 4-fold lower and between U73 to U86 and R3 to U95 was 2-fold lower than the rest of the genome. Quantitative PCR (qPCR) analysis of origin and U32 loci confirmed the deep-sequencing data, demonstrating a range between 1 and 10× higher copy numbers of the origin than the U32 housekeeping gene (Fig. 2). Of note, an early-passage GS strain had equal copy numbers of the origin and U32 genes, while the later-passage GS-HSB2 strain had a 5.8-fold higher copy number at the origin, consistent with what had been described previously in Z29 (31). Both HHV-6A GS and HHV-6B Z29 strains that did not have large deletions or rearrangements outside the origin repeat had direct-repeat copy numbers approximately two-thirds that of the average coverage of the unique region when aligned with a reference genome with two direct repeats (Table 1). No differences in the lengths of direct-repeat regions were apparent in our strains, as was found in the high-passage-number HHV-6A strain U1102 (35).

**TABLE 1:** Summary of HHV-6A/B strains sequenced in this study

Species	Strain	Cell line	No. of copies/ml			No. of trimmed reads	No. of HHV-6 reads	Unique/DR coverage	Accession no.
			U32	Origin	U95				
HHV-6A	GS	HSB2	3.30E9	1.90E10	1.80E9	2,091,792	47,508	54×/36×	MF994822
	GS-early	CBMC	3.30E4	3.40E4		308,104	66,878	60×/37×	KC465951^*a*^
	DA	HSB2	2.60E9	2.40E10		3,787,217	30,508	31×/19×	MF994820
	CO-1	HSB2	3.60E9	4.00E10	1.70E9	1,913,207	164,835	165×/107×	MF994815
	CO-2	HSB2	2.60E9	3.20E10	1.00E9	1,278,582	118,403	135×/84×	MF994816
	CO-3	HSB2	5.60E8	5.20E9	2.90E8	896,950	54,464	65×/39×	MF994817
	CO-4	HSB2	5.70E8	6.20E9	5.00E6	6,692,042	42,596	35×/34×	MF994818
	CO-7	HSB2	2.20E8	4.20E9	1.40E6	10,887,157	54,046	43×/42×	MF994819
	SIE	PHA-stimulated CBMC	1.20E10	1.10E10		1,015,200	49,307	63×/36×	MF994828
	ABI-HHV6A (GS)	Unknown	6.41E5	8.98E6		1,644,798	225,145	165×/66×	MF994813
HHV-6B	Z29	SupT1	2.00E6	1.80E7		2,436,488	62,770	90×/52×	MF994829
	HST	MT4	1.10E7	1.50E7		10,666,502	28,251	33×/19×	MF994824
	HST	PHA-stimulated CBMC	1.70E9	1.10E9		1,155,336	29,365	39×/25×	MF994823
	KYO	PHA-stimulated CBMC	7.00E8	8.60E8		949,314	28,626	38×/25×	MF994825
	ENO	PHA-stimulated CBMC	3.30E9	2.00E9		1,116,480	39,004	51×/32×	MF994821
	MAR	PHA-stimulated CBMC	2.30E8	2.70E8		801,650	26,594	34×/21×	MF994826
	NAK	PHA-stimulated CBMC	3.60E6	5.40E6		5,617,132	42,955	44×/33×	MF994827
	ABI-HHV6B (Z29)	Unknown	1.50E5	1.20E6		1,200,052	112,632	104×/100×	MF994814

a Sequenced previously (21); reads received from L. Flamand.

**FIG 3:**
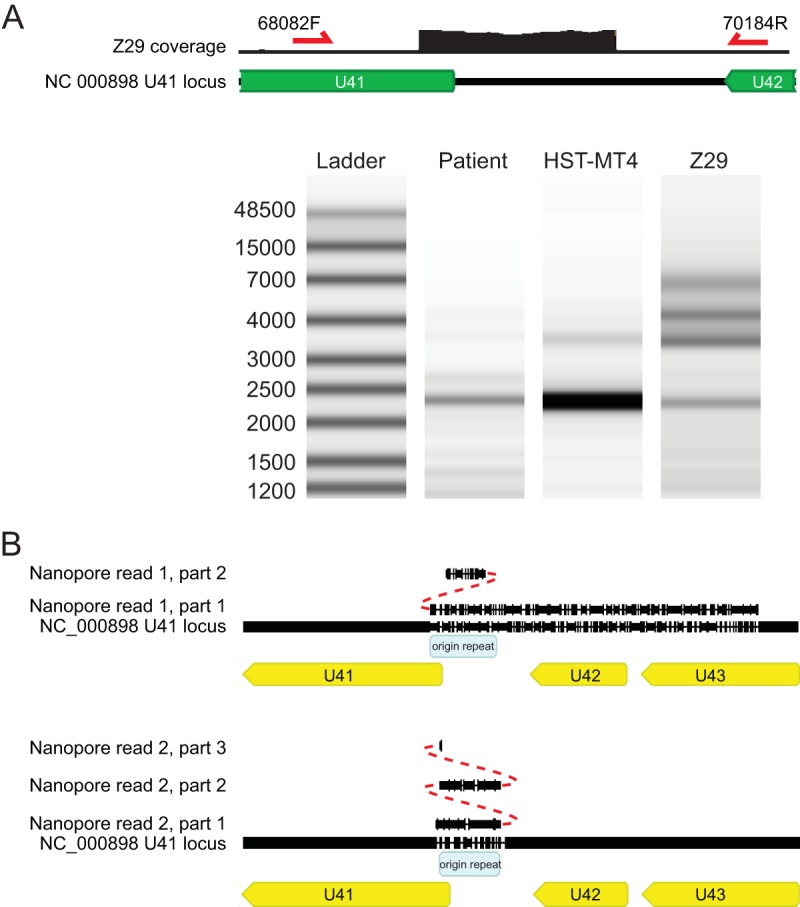
Validation of the Z29 origin tandem repeat with PCR and nanopore sequencing. (A) Electrophoresis analysis of PCR across the tandem repeat in the origin of the patient sample, HST (from the MT4 cell line), and Z29 strains. (B) Amplification-free nanopore sequencing of Z29 strains yielded two nanopore reads that aligned across the origin tandem repeat and carried at least 2 and 3 copies of the repeat. Indels in the read account for the gaps in the read and the reference genome. No reads that spanned both ends of the tandem repeat were recovered.

Previous analysis of 125 HHV-6B genomes obtained from clinical specimens revealed no tandem repeats across the origin of replication (20). To confirm the origin tandem repeat present in HHV-6B strain Z29, we performed PCR amplification and fragment analysis by gel electrophoresis across the sequence tandem repeat with two separate primer sets. As a control, we performed PCR across the origin of replication in an HHV-6B PCR-positive patient specimen. The patient specimen demonstrated the presence of a single copy of the locus, while the Z29 strain contained an amplification ladder, consistent with a population of virus with different numbers of multiple tandem repeats present at the locus (Fig. 3A). We also performed amplification-free long-read nanopore sequencing on DNA extracted from HHV-6B strain Z29 in culture. Across 6,369 nanopore reads with an average read length of 3,235 nucleotides, we recovered two reads that contained more than one copy of the origin tandem repeat (Fig. 3B). One read contained three tandem repeats of the origin repeat, while the other contained two repeats. Neither read spanned the entire tandem repeat, consistent with the longer complex repeats previously observed by Southern blotting of multiple Z29 strains in culture (31).

### Interspecies recombination between HHV-6A and HHV-6B in strain DA.

**FIG 4:**
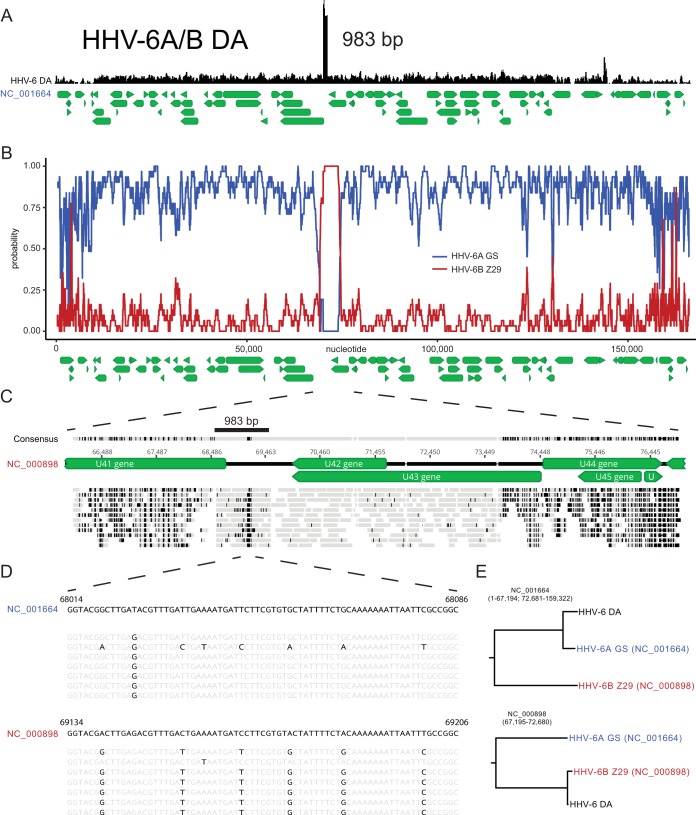
HHV-6A strain DA shows genomic evidence of interspecies recombination between HHV-6A and HHV-6B strains. (A) Tandem repeat of the origin for strain DA showing a Z29-like length of 983 bp. Gene annotations are depicted in green below the genome. (B) RDP4 scan recombination analysis demonstrating two recombination breakpoints at nucleotides 67194 and 72681 of the HHV-6A DA genome. (C) Mapping of a selection of reads to the HHV-6B Z29 reference genome at the U41 locus, with reads depicted in gray and disagreements with the HHV-6B Z29 reference genome highlighted in black. (D) Nucleotide sequence of the 61-bp region of the tandem repeat that most closely matches the HHV-6A reference genome (NC_001664), with base agreements depicted in gray and disagreements with the HHV-6A and HHV-6B reference genomes highlighted in black. (E) Phylogenetic tree analysis of the recombination region supports the HHV-6B-like nature of the U41 locus.

Whole-genome sequencing of the DA strain revealed a hybrid genome indicative of interspecies recombination between HHV-6A and HHV-6B strains. The DA strain genome overall showed closer sequence identity to HHV-6A than to HHV-6B strains but included an oriLyt repeat that measured the exact length of the Z29 repeat at 983 bp (Fig. 4A). The DA strain U38 gene matched with perfect identity that of the HHV-6A GS strain by BLASTN analysis. Of note, the DA strain origin-binding protein U73 gene more closely matched HHV-6A U73 than HHV-6B U73 (99.5% versus 97.1% pairwise nucleotide identity to HHV-6A and HHV-6B reference strain genomes, respectively).

Analysis of the DA strain sequence yielded consensus recombination breakpoints at bp 67314 and bp 72667 of the HHV-6A U1102 reference genome (NC_001664) that were detected by six of seven recombination analysis programs (RDP4, GENECONV, Bootscan, MaxChi, Chimaera, and 3Seq) (Fig. 4B). Reads mapped with near identity along a 5.5-kb region of the HHV-6B Z29 reference genome (NC_000898) between the 5′ end of U41 and the 3′ end of U43 (bp 68535 to 73824 of NC_000898) (Fig. 4C). Only a 61-bp fragment with an oriLyt repeat had 6 variant sites compared to HHV-6B sequences and matched identically the HHV-6A sequences (Fig. 4D). These sequences were just 3′ from the end of the minimal origin of DNA replication annotated in the HHV-6A reference genome. The length of the HHV-6B sequence present in the DA strain is likely considerably greater than the 5.5-kb difference illustrated on the HHV-6B Z29 reference genome (NC_000898) in Fig. 4C, due to the presence of the oriLyt repeat. Within the unique region, strain DA contained 66 single nucleotide variants relative to HHV-6 strain GS (KC465951) between genes U3 and U41, U44 and U85, and U94 and U100 outside the repeat regions and the oriLyt repeat.

### Large deletions in U12 to U24 genes and U94 and U95 genes from two laboratory-adapted HHV-6A strains from collagen vascular disease.

**FIG 5:**
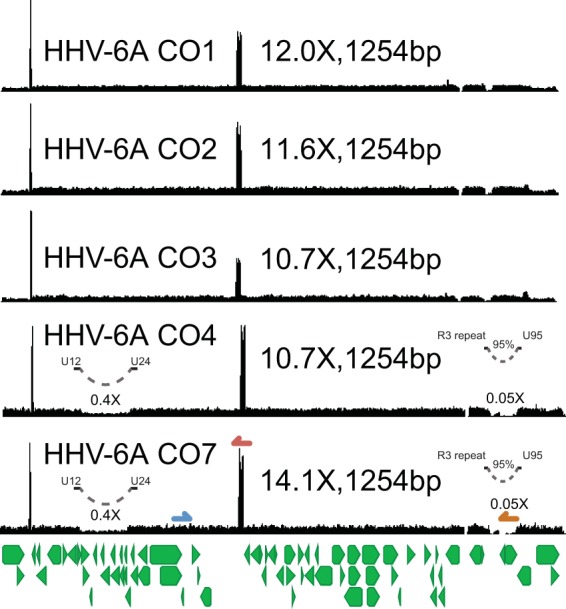
HHV-6A CO strains from patients with collagen vascular diseases show several copy number differences. (A) Coverage maps from five HHV-6A strains isolated from different patients with collagen vascular diseases (36). These strains all showed similar heterogeneous tandem repeats that gave a mode length of 1,254 bp. Strains CO4 and CO7, which were passaged >40 times in immortalized HSB2 cell lines, also demonstrated 60% and 95% lower coverage in the U12-to-U24 and U94-to-U95 regions, respectively. Of note, these two strains also shared identical sequences and minor allele distributions, consistent with being the same strain. Gene annotations are depicted in green below the genome. U32 (blue), U41/origin (red), and U95 (orange) primer sites are illustrated on the coverage map for strain CO7.

**FIG 6:**
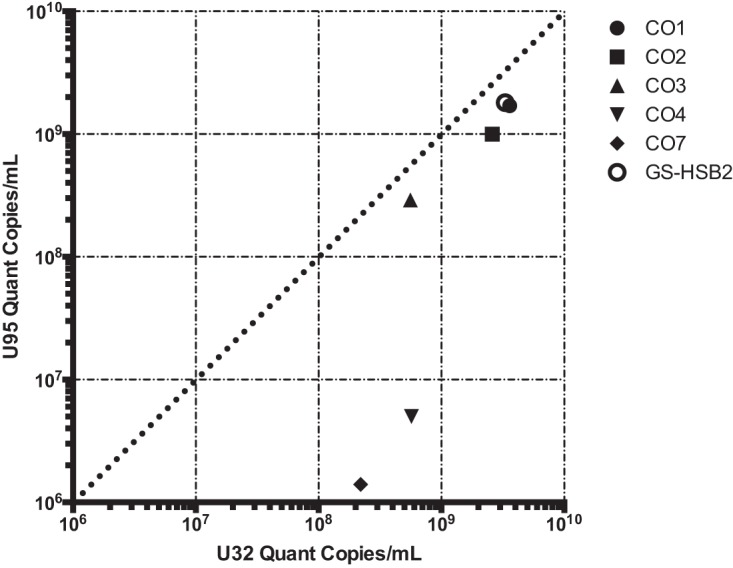
qPCR analysis of genomes from the HHV-6A strains at the U32 and U95 loci confirming relative copy number estimates from deep-sequencing data. The plotted quantities are listed in Table 1.

Five HHV-6A strain isolates from patients with collagen vascular disease also grew to high copy numbers in HSB2 cells. Isolates CO1, CO2, and CO3 were cultured in primary peripheral blood lymphocytes for 17 to 21 days and then in HSB2 cells for 4 to 5 days, while isolates CO4 and CO7 were cultured for 2 days in primary cells and 42 to 46 days in HSB2 cells (36). The five CO isolates were highly similar, with an average pairwise nucleotide identity of >99.9%, while all five isolates most closely aligned with HHV-6A isolate GS (KJ123690.1; 99.2% pairwise nucleotide identity). Both isolates CO4 and CO7 had 60% decreased coverage in a 13.5-kb region from U12 to U24 relative to the CO1 to CO3 isolates (Fig. 5). Most notably, both isolates CO4 and CO7 also had 95% lower coverage over a 4.9-kb region covering genes U94 and U95, than the CO1 to CO3 isolates. Equivalent relative copy number estimates for both the U95 locus and the origin of replication were recovered by qPCR (Fig. 2 and 6). Isolates CO4 and CO7 both had equivalent mixed variant allele frequencies at 25 loci based on read mapping to the unique region of the HHV-6A reference genome (NC_001664) (Table 2). No other variants were isolated between the HHV-6A CO4 and CO7 strains, suggesting the strains are identical.

**TABLE 2:** Loci with variant allele frequencies between 25 and 75% at nucleotides 8500 to 150000 of NC_001664^*a*^

Nucleotide no.	Virus strain	Sequencing depth	Variant frequency (%)	Polymorphism type^*b*^	Gene	Change	Amino acid change	CDS codon no.	Protein effect
15942	HHV-6 CO4	22	31.80	SNP (transition)		C→T			
15942	HHV-6 CO7	40	45.00	SNP (transition)		C→T			
16978	HHV-6 CO4	35	40.00	SNP (transversion)	U7	G→T		38	None
16978	HHV-6 CO7	51	43.10	SNP (transversion)	U7	G→T		38	None
18687	HHV-6 CO4	37	48.60	SNP (transition)	U10	G→A	D→N	362	Substitution
18687	HHV-6 CO7	46	50.00	SNP (transition)	U10	G→A	D→N	362	Substitution
18877	HHV-6 CO4	33	48.50	SNP (transversion)	U10	C→A	P→H	425	Substitution
18877	HHV-6 CO7	32	59.40	SNP (transversion)	U10	C→A	P→H	425	Substitution
19258	HHV-6 CO7	30	50.00	SNP (transition)	U11	T→C	D→G	774	Substitution
19258	HHV-6 CO4	26	50.00	SNP (transition)	U11	T→C	D→G	774	Substitution
29716	HHV-6 CO7	19	36.80	SNP (transversion)	U19	T→A	K→I	368	Substitution
29716	HHV-6 CO4	15	53.30	SNP (transversion)	U19	T→A	K→I	368	Substitution
36965	HHV-6 CO7	47	34.00	Insertion (tandem repeat)	U26	(A)8→(A)9		282	Frame shift
36965	HHV-6 CO4	27	37.00	Insertion (tandem repeat)	U26	(A)8→(A)9		282	Frame shift
40589	HHV-6 CO4	42	31.00	SNP (transition)	U28	C→T		282	None
40589	HHV-6 CO7	42	50.00	SNP (transition)	U28	C→T		282	None
58704	HHV-6 CO4	21	33.30	SNP (transition)	U38	G→A		295	None
69117	HHV-6 CO4	33	45.50	SNP (transition)	U42	A→G		494	None
69117	HHV-6 CO7	58	50.00	SNP (transition)	U42	A→G		494	None
69133	HHV-6 CO7	60	48.30	SNP (transition)	U42	A→G	V→A	489	Substitution
69133	HHV-6 CO4	35	48.60	SNP (transition)	U42	A→G	V→A	489	Substitution
72012	HHV-6 CO7	49	51.00	SNP (transition)	U43	C→T	C→Y	465	Substitution
72012	HHV-6 CO4	39	56.40	SNP (transition)	U43	C→T	C→Y	465	Substitution
74768	HHV-6 CO4	42	66.70	SNP (transition)	U45	T→C	N→D	151	Substitution
74768	HHV-6 CO7	32	68.80	SNP (transition)	U45	T→C	N→D	151	Substitution
92258	HHV-6 CO7	45	33.30	SNP (transition)	U57	C→T	C→Y	552	Substitution
92258	HHV-6 CO4	30	36.70	SNP (transition)	U57	C→T	C→Y	552	Substitution
106203	HHV-6 CO4	21	33.30	SNP (transition)	U70	G→A		214	None
106203	HHV-6 CO7	45	48.90	SNP (transition)	U70	G→A		214	None
106566	HHV-6 CO4	32	37.50	SNP (transversion)	U70	T→A	D→E	335	Substitution
106566	HHV-6 CO7	39	59.00	SNP (transversion)	U70	T→A	D→E	335	Substitution
107612	HHV-6 CO4	28	57.10	SNP (transition)	U72	G→A	T→I	234	Substitution
107612	HHV-6 CO7	40	60.00	SNP (transition)	U72	G→A	T→I	234	Substitution
109716	HHV-6 CO7	37	43.20	SNP (transition)	U73	G→A		464	None
109716	HHV-6 CO4	40	55.00	SNP (transition)	U73	G→A		464	None
110489	HHV-6 CO4	33	42.40	SNP (transition)	U73	A→G	E→G	722	Substitution
110489	HHV-6 CO7	46	43.50	SNP (transition)	U73	A→G	E→G	722	Substitution
112286	HHV-6 CO7	44	40.90	SNP (transition)	U74	T→C	Y→H	551	Substitution
112286	HHV-6 CO4	42	61.90	SNP (transition)	U74	T→C	Y→H	551	Substitution
113466	HHV-6 CO4	25	60.00	SNP (transversion)	U76	G→T	H→N	598	Substitution
113466	HHV-6 CO7	46	65.20	SNP (transversion)	U76	G→T	H→N	598	Substitution
121571	HHV-6 CO4	11	63.60	Deletion	U79	-T		410	Frame Shift
121571	HHV-6 CO7	16	68.80	Deletion	U79	-T		410	Frame Shift
123584	HHV-6 CO4	22	36.40	SNP (transversion)	U83	A→T		19	None
123584	HHV-6 CO7	42	42.90	SNP (transversion)	U83	A→T		19	None
123591	HHV-6 CO4	20	30.00	SNP (transition)	U83	T→C	S→P	22	Substitution
123591	HHV-6 CO7	44	45.50	SNP (transition)	U83	T→C	S→P	22	Substitution

a Matched loci with variant allele frequencies between 30 and 75%, coverage of >10× at nucleotides 8,500 to 150000 of NC_001664 for HHV-6A strains CO-4 and CO-7.

b SNP, single nucleotide polymorphism.

### Reduced-passage HHV-6A/B strains do not contain large tandem duplications or deletions.

**FIG 7:**
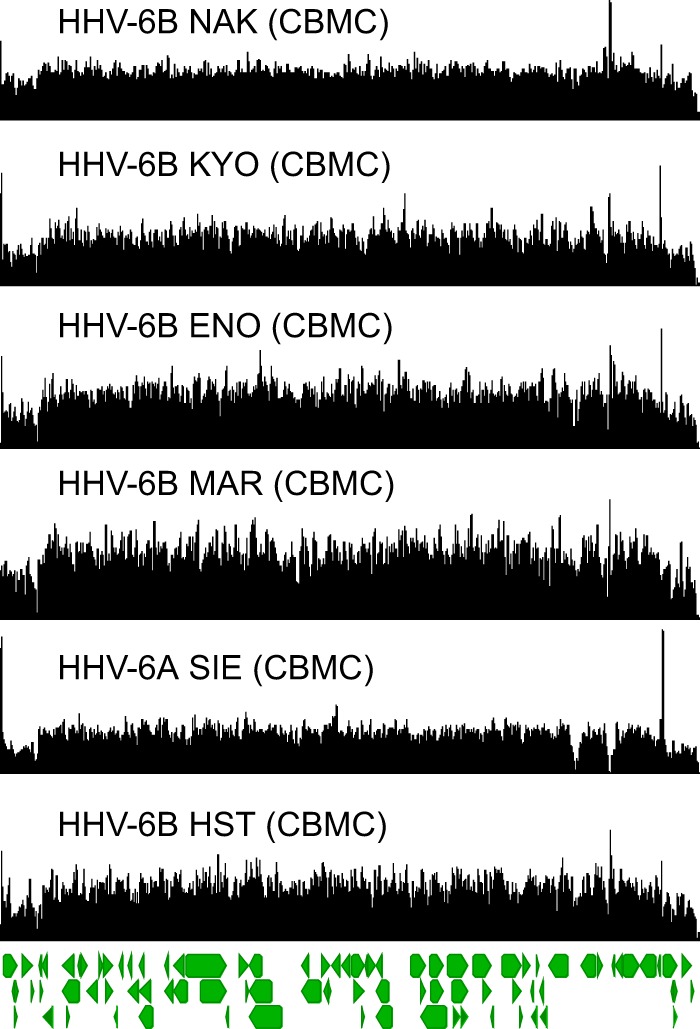
HHV-6A/B strains sequenced from PHA-activated cord blood mononuclear cells revealed no major copy number differences. Coverage maps from four HHV-6B strains from Japan (NAK, KYO, ENO, and HST), one HHV-6B strain from France (MAR), and one HHV-6A strain from Ivory Coast (SIE) are shown. The major copy number difference in these strains is the reduced coverage in the direct-repeat region, likely due to sequencing of HHV-6A/B strains with only one direct repeat. The direct-repeat coverage difference was present in most of the strains sequenced in this study (Table 1).

To find the best standard for HHV-6A/B clinical testing and to better understand the source of the origin tandem repeat, we sequenced four HHV-6B strains and one HHV-6A strain that were isolated from phytohemagglutinin (PHA)-stimulated cord blood mononuclear cells (CBMC). Four HHV-6B strains (HST, ENO, KYO, and NAK) were isolated from Japanese exanthem subitum patients, one HHV-6B strain (MAR) was isolated from an asymptomatic French child, and one HHV-6A strain (SIE) was isolated from an Ivory Coast patient with adult T-cell leukemia (37–39). Interestingly, all six strains contained minimal levels of copy number heterogeneity, with an average coefficient of variation of coverage in the unique region of 21.0% compared with 119% averaged over the HHV-6A GS and HHV-6B Z29 type strains (Fig. 7). The lack of origin amplification in these CBMC-passaged strains was also confirmed by qPCR (Fig. 2). Without the tandem repeat in the origin, the only source of copy number differences in the cord blood mononuclear cell-passaged strains was direct-repeat coverage at approximately two-thirds of that in the unique region when aligned with a reference genome with two direct repeats, consistent with active replication and sequencing of mostly single DRs containing HHV-6A/B genomes (Fig. 7 and Table 1) (40, 41). Of note, the decreased coverage in the direct-repeat region relative to the unique region was present in all the strains sequenced in this study.

## DISCUSSION

We showed the presence of copy number heterogeneity at multiple genomic loci across HHV-6A and HHV-6B culture isolates that are used as reference material for clinical assay development and normalization, as well as basic-science virology work. The most prominent copy number difference was a large tandem repeat in the origin of replication that was present in both HHV-6A and HHV-6B strains. The presence of large copy number differences in HHV-6A and HHV-6B strains was associated with passaging in immortalized cell lines, as strains passaged in primary cell lines and cord blood mononuclear cells did not carry genomic duplications and deletions and early-passage virus contained fewer tandem repeats than late-passage virus.

Recent analysis of 125 HHV-6B genomes and 10 iciHHV-6A sequences spanning the U41 origin region revealed no large tandem repeats in the origin of replication among clinical isolates (20). Previous work had shown that a large heterogeneous tandem repeat present at the oriLyt in HHV-6B Z29 strains associated with higher passage numbers conferred a growth advantage *in vitro* and that tandem HHV-6B origin elements were more efficiently replicated *in vitro* (31, 32). Our data show that this is a general feature of HHV-6A/B and that a heterogeneous larger tandem repeat is also present in multiple laboratory-adapted HHV-6A strains at the oriLyt. We showed that increased culture passage was associated with reduced copy numbers of two additional large genomic loci in HHV-6A CO strains. Unfortunately, increased culture passage numbers and passage in immortalized cell lines were correlated in our study, making it difficult to pinpoint the exact role of passage number versus cell line type. Based on the data from the early-passage strain of GS and previous work done in Z29, the data are most consistent with increased copy numbers being associated with increased passage numbers rather than passage in immortalized cell lines (31). We also hypothesize that an increased copy number of the origin confers a growth advantage for HHV-6A/B, as it arose independently in both HHV-6A and HHV-6B type strains and is associated and maintained with increasing passage numbers, although more experiments are required to formally characterize the mechanism of this phenomenon. With the discovery of the origin repeats in HHV-6A strain GS, transgenic experiments can now be performed in the HHV-6A bacterial artificial chromosome system, in addition to plasmid-based replicon experiments used to characterize origins (41).

Copy number variability at multiple genomic loci was also reflected in HHV-6A/B reference material that is used to normalize quantitative values for clinical assay development. It is intriguing to speculate whether HHV-6A/B strains missing tens of kilobases from the U12-to-U20/U24 or U73-to-R3 regions are replication competent. No doubt these viruses were grown at high multiplicities of infection, and it is highly likely virus with deletions could be complemented with intact virus. Nevertheless, the U12-to-U20 and U12-to-U24 deletion viruses (which also include 4 C-terminal residues of U25) in the HHV-6B Z29 ABI reference material and CO4-7 strains are notable, as none of these genes have been shown to be essential for HHV-6A/B replication (16, 42, 43). The U20 to U24 genes are Roseolovirus-specific immunomodulatory genes and have been shown to be dispensable for viral replication *in vitro* (42, 43). By analogy to the human cytomegalovirus (HCMV) genome, the HHV-6A U14 to U17 proteins are HCMV UL25-like proteins, which are not required for HCMV replication (44). The boundaries of the deletion may not be due entirely to chance, as U11 is an essential gene for HHV-6A replication (45). HHV-6A with U94 deleted is replication competent, albeit with a significant growth disadvantage when tested *in vitro* (46). Replication of the origin may help rescue such replication defects, although it is still unclear why such large deletions covering genes required for replication are recovered in high-passage-number material.

The U73-to-R3 deletion is more difficult to explain, as many of the genes that are encompassed by the deletion are required for replication in other herpesviruses. We note that repeated library preparations with different library preparation technologies (Nextera XT transposase and Kapa HyperPlus nuclease) gave the same rearrangement. As commercial material, its passage history is not as completely clear. Although not previously formally tested in HHV-6A/B, the helicase-primase complex (containing U74 and U77 in HHV-6A/B) is essential for HSV and HCMV replication, as is the HSV UL9 gene, encoding the homolog of the HHV-6A/B U73 origin-binding protein (44). The HSV UL2 homolog of the HHV-6A/B U75 uracil deglycosylase is not required for replication *in vitro* (47). HHV-6A/B U82 encodes glycoprotein L, which is required in HSV and HCMV replication (44). The HCMV homologs (UL123 and UL124) of HHV-6A/B U90 and U91 are not required for viral replication (44).

Interestingly, no low-passage-number HHV-6A/B isolate grown in cord blood mononuclear cells showed tandem repeats, despite growing to high titer. These low-passage-number cord blood isolates may provide the best material for HHV-6A/B standard development. Instead, the isolates showed direct-repeat copy numbers that were approximately 60 to 70% that of the unique region. This copy number difference was present in the majority of strains sequenced in this study and suggests that the majority of HHV-6A/B strains sequenced contained only one direct-repeat region repeat, which has previously been seen in HHV-6A/B replication (35, 41, 48, 49).

We also showed the first genomic evidence of interspecies recombination between HHV-6A and HHV-6B strains along a >5.5-kb segment containing the oriLyt and the U42 gene, as well as portions of the U41 and U43 genes. Interspecies recombination is a relatively common feature of the alphaherpesviruses HSV-1 and HSV-2 but has not been described for any other human herpesviruses (17). The origin of the DA recombinant is not entirely clear. Our sequencing results for DA strain recombination are most consistent with a model in which cocultivation of an HHV-6A strain with a laboratory-adapted HHV-6B Z29 strain containing an oriLyt repeat resulted in recombination between the two strains. Strains GS and Z29 were both cultured in cord blood lymphocytes in the laboratory at the same time strain DA was being cultured. The DA strain oriLyt repeat is the exact same length as that of the Z29 strain. However, the recombinant strain also showed unique HHV-6A-like sequence in its HHV-6B oriLyt tandem repeat. Although this sequence fell outside the minimal origin of replication, it is possible that the oriLyt tandem-repeat sequence may have evolved to more effectively interact with HHV-6A replication proteins contained in the rest of the genome. No specific HHV-6B-like sequences were found in the DA strain U73 origin-binding protein to indicate reciprocal U73 evolution to match the HHV-6B-like origin sequences. Despite the high sequence similarity between HHV-6A and HHV-6B, recent analysis of 130 HHV-6B genomes from clinical and iciHHV-6B isolates revealed no evidence of interspecies recombination but widespread intraspecies recombination (20). We note that the ability of HSV-1 and HSV-2 to recombine was first noted *in vitro*, before the discovery of clinical strain recombinants, which are widespread (17, 50, 51). Whether interspecies recombination will ever be detected in clinical strains of HHV-6A/B remains to be seen.

Previous work from our group demonstrated the loss of almost one-third of the BK and JC polyomavirus genomes in up to 90% of viral species present in multiple viral stocks, including a WHO international standard, likely due to viral passage in simian virus 40 (SV40) T-antigen-immortalized cell lines (33, 34). While clinical PCR tests for HHV-6A/B are unlikely to target the oriLyt region, normalization of quantitative clinical HHV-6A/B testing to any of the loci found here at increased or decreased copy numbers could affect quantitation, not least because multiple viral populations were present in many of the reference materials tested. Based on the deletions detected in HHV-6A/B clinical reference materials, primers targeting genes between U25 and U40 and between U43 and U73 would be expected to show no copy number differences. We note that many of the published diagnostic primers target U32 and U67, which both fall within this range of core genes (52). Efforts are under way at the National Institute for Biological Standards and Controls in the United Kingdom to prepare WHO international standard material for both HHV-6A and 6B. We continue to recommend the use of next-generation sequencing to obtain genome-wide single nucleotide resolution copy number measurements in order to validate viral reference materials used in clinical virology and basic-science laboratories around the world.

## MATERIALS AND METHODS

### HHV-6A/B reference strains and DNA materials.

HHV-6A/B culture isolates were obtained from the HHV-6 Foundation. The original HHV-6A isolate GS was first isolated at the National Cancer Institute (NCI), NIH, in 1986 from an AIDS patient (1). The HHV-6A GS strain was grown in HSB2, which is a human T-cell leukemic cell line derived from the peripheral blood of a child. The GS early-passage isolate obtained from the HHV-6 Foundation is a low-passage HHV-6A GS isolate that was passaged only 4 times in CBMC. The GS early-passage isolate was sequenced in 2013 after a brief expansion in CBMC, and sequencing reads were obtained from L. Flamand (54). The HHV-6A DA strain was isolated at the NCI from a patient with chronic fatigue syndrome and was grown in the HSB2 cell line. The HHV-6A CO strains CO1, CO2, CO3, CO4, and CO7 were isolated from patients with collagen vascular diseases, including systemic lupus erythematosus, atypical polyclonal lymphoproliferation, rheumatoid arthritis, and unclassified collagen vascular disease (33). The HHV-6A SIE strain was isolated from an HIV-positive leukemia patient from the Ivory Coast and grown in PHA-stimulated CBMC. The HHV-6B strains HST, KYO, ENO, and NAK were isolated from Japanese patients with exanthema subitum in 1988 (37). The HHV-6B MAR strain was originally obtained from an HIV-negative child born to an HIV-positive mother and has been cultured in CBMC (38). HHV-6B strain Z29 was originally isolated from an AIDS patient from Zaire and obtained from the HHV-6 Foundation stock deposited at the NIH AIDS repository and was grown in the SupT1 cell line. HHV-6A/B standards comprising quantitated viral DNA from HHV-6A strain GS (08-945-250) and HHV-6B strain Z29 (08-923-00) were purchased from Advanced Biotechnology Inc. The strains sequenced in this study are listed in Table 1.

### Illumina sequencing library preparation.

DNA was extracted from culture isolates using the Zymo viral DNA kit. DNA-sequencing libraries were prepared from 50 ng of genomic DNA using quarter volumes of the Kapa HyperPrep kit with 7 min of fragmentation time and 12 cycles of dual-indexed TruSeq adapter PCR (20). The libraries were sequenced on 2 × 300-bp, 1 × 190-bp, and/or 1 × 192-bp runs on an Illumina MiSeq. The sequences were quality and adapter trimmed using BBDuk (http://jgi.doe.gov/data-and-tools/bbtools/) and *de novo* assembled using SPAdes (58), and contigs were aligned with reference HHV-6A (NC_001664) and HHV-6B (NC_000898) genomes and visualized using Geneious v9.1. Read mapping for copy number analysis was performed against the HHV-6A (NC_001664) and HHV-6B (NC_000898) reference genomes, using the Geneious read mapper with 10% allowed gaps per read, word length of 18, and 20% maximum mismatches per read and with structural variant, insertion, and gap finding allowed.

### qPCR confirmation.

Quantitative PCR to estimate copy numbers between the origin of replication and the HHV-6A/B U32 locus was performed in 20-μl reaction mixtures using the SsoAdvanced Universal SYBR green SuperMix (Bio-Rad). Three 10-fold dilutions of DNA template from HHV-6A/B strains were tested using quantU32 F-R and species-specific origin primers (Table 3) with cycling conditions of 95°C for 30 s and 40 cycles of 95°C for 5 s and 60°C for 30 s. For the U95 deletion in the CO strains, CO4-142970F and CO-143228R primers (Table 3) were used under the same cycling conditions with SsoAdvanced Universal SYBR green SuperMix, and the U32-targeting qPCR was performed as described above. Absolute quantitation of viral strains was performed using six 10-fold dilutions of an HHV-6 U32 amplicon cloned into a plasmid that was quantitated using digital PCR (55). Quantitation of U95 and origin qPCR were performed based on U32 absolute quantitation using six 10-fold dilutions of viral strains without deletions or amplifications present in the sequencing data (GS-HSB2 for U95 and HST-CBMC for origin). Quantities are listed in Table 1.

**TABLE 3:** Primer pairs used in this study

Primer	Sequence
PCR of HHV-6B	
HHV6_68082F	GTTTCACTTAACGCAGGCAG
HHV6_70190R	GAAAACGTCAACTCTAAACATGAAAG
qPCR	
HHV6_CO4-142970F	GGCAACAAATTCTCAATATGGAT
HHV6_CO4-143228R	ATTTGATTGTTCATGTCTTCCG
HHV6A-origin-F	CGAGGGCGTGGCGTTTAC
HHV6B-origin-F	CGAAGGCGTGGCGTTTAC
HHV6-common-origin-R	GCTCGCAGCCTTTTTAAATCC
HHV6-quantU32-F	CTTTCATCAATCACTCCCTGTTTTT
HHV6-quantU32-R	GCGTTCCGACTCGATTTGATA
HHV6-U32-probe	TGTTCAGTCTATGCAGCGCCAGCATTC

### PCR and electrophoretic analysis.

PCR across the origin tandem repeat was performed using 1 ng of template genomic DNA in 20-μl total volume reaction mixtures using 10 pmol of each primer and the NEB HiFi Q5 DNA polymerase according to the manufacturer's instructions. PCR primer sequences are listed in Table 3. PCR mixtures were analyzed with the Genomic DNA ScreenTape assay on an Agilent 4200 TapeStation to visualize the electrophoretic mobility of the PCR products.

### Amplification-free nanopore sequencing.

Nanopore libraries were created using the SQK-RAD002 kit tagmentation library preparation with 100 ng of input total genomic DNA from the HHV-6B Z29 strain cultured in SupT1 cells (Oxford Nanopore Technologies). Amplification-free tagmented libraries were run according to Oxford Nanopore protocols v1.3.24 on a singular Mk1 (R9.4) FLO-MIN106 flow cell. Nanopore reads were mapped to the HHV-6B Z29 reference genome (NC_000898) using the LASTZ and Geneious aligners to screen for origin-containing reads (56, 57).

### Accession number(s).

The sequences from this study are available in NCBI GenBank (accession numbers MF994813 to MF994829) and associated with NCBI BioProject 338014.
